# Enablers and barriers to physical activity in overweight and obese pregnant women: an analysis informed by the theoretical domains framework and COM-B model

**DOI:** 10.1186/s12884-018-1816-z

**Published:** 2018-05-21

**Authors:** C. Flannery, S. McHugh, A. E. Anaba, E. Clifford, M. O’Riordan, L. C. Kenny, F. M. McAuliffe, P. M. Kearney, M. Byrne

**Affiliations:** 1 Health Behaviour Change Research Group, School of Psychology, National University of Ireland, Galway, Ireland; 2 School of Public Health, University College Cork, Cork, Ireland; 3 Department of Nutrition & Dietetics, South Infirmary Victoria University Hospital, Cork, Ireland; 4 Department Obstetrics and Gynaecology, University College Cork, Cork, Ireland; 5 Department of Women’s and Children’s Health, Faculty of Health and Life Sciences, University of Liverpool, Liverpool, UK; 6 UCD Perinatal Research Centre, School of Medicine, University College Dublin, National Maternity Hospital, Dublin, Ireland

**Keywords:** Overweight, Obesity, Pregnant women, Maternal health, Physical activity, Theoretical domains framework, COM-B model, Behaviour change wheel

## Abstract

**Background:**

Obesity during pregnancy is associated with increased risk of gestational diabetes mellitus (GDM) and other complications. Physical activity is a modifiable lifestyle factor that may help to prevent these complications but many women reduce their physical activity levels during pregnancy. Interventions targeting physical activity in pregnancy are on-going but few identify the underlying behaviour change mechanisms by which the intervention is expected to work. To enhance intervention effectiveness, recent tools in behavioural science such as the Theoretical Domains Framework (TDF) and COM-B model (capability, opportunity, motivation and behaviour) have been employed to understand behaviours for intervention development. Using these behaviour change methods, this study aimed to identify the enablers and barriers to physical activity in overweight and obese pregnant women.

**Methods:**

Semi-structured interviews were conducted with a purposive sample of overweight and obese women at different stages of pregnancy attending a public antenatal clinic in a large academic maternity hospital in Cork, Ireland. Interviews were recorded and transcribed into NVivo V.10 software. Data analysis followed the framework approach, drawing on the TDF and the COM-B model.

**Results:**

Twenty one themes were identified and these mapped directly on to the COM-B model of behaviour change and ten of the TDF domains. Having the social opportunity to engage in physical activity was identified as an enabler; pregnant women suggested being active was easier when supported by their partners. Knowledge was a commonly reported barrier with women lacking information on safe activities during pregnancy and describing the information received from their midwife as ‘limited’. Having the physical capability and physical opportunity to carry out physical activity were also identified as barriers; experiencing pain, a lack of time, having other children, and working prevented women from being active.

**Conclusion:**

A wide range of barriers and enablers were identified which influenced women’s capability, motivation and opportunity to engage in physical activity with “knowledge” as the most commonly reported barrier. This study is a theoretical starting point in making a ‘behavioural diagnoses’ and the results will be used to inform the development of an intervention to increase physical activity levels among overweight and obese pregnant women.

**Electronic supplementary material:**

The online version of this article (10.1186/s12884-018-1816-z) contains supplementary material, which is available to authorized users.

## Background

Recent studies identify increasing trends in maternal obesity worldwide and associated complications such as gestational diabetes mellitus (GDM) [1–3]. Maternal obesity also has adverse neonatal outcomes, such as macrosomia [4] and offspring born to obese women are more likely to develop obesity, type 2 diabetes, cardiovascular disease and cancer in later life [5]. A recent systematic review identified maternal pre-pregnancy overweight as a significant risk factor for childhood overweight [6]. Children of mothers who were overweight before pregnancy were 1.37 times more likely to be overweight at 3 years of age than children of normal weight parents [7]. These trends and risks have increased interest in antenatal interventions which focus on women’s eating, physical activity, their impact on gestational weight gain and GDM [8–10]. Strong evidence exists on the benefits associated with physical activity during pregnancy including an increase in functional mobility and a reduction in nausea and vomiting [11, 12]. Higher levels of physical activity before pregnancy or in early pregnancy also significantly lowers the risk of developing GDM [13]. A recent meta-analysis reported that antenatal physical activity in women of any body mass index led to a small reduction in offspring birth weight [14]. It is possible that this modest reduction in birth weight in offspring of overweight and obese women may be beneficial in reducing the long-term obesity risk [14, 15]. Furthermore, behavioural changes made during pregnancy may continue after childbirth and possibly throughout the woman’s life [16] which in turn may have positive effects on child physical activity levels [17].

Despite these benefits, women’s physical activity levels often reduce or cease during pregnancy [18]. Similar to Health Service Executive (HSE) recommendations in Ireland, the American Congress of Obstetricians and Gynaecologists (ACOG) and the Royal College of Obstetricians and Gynaecologists (RCOG), UK, recommend 30 min of daily moderate intensity physical activity for pregnant women [19–22]. Previous studies, carried out in different countries, reported low rates of physical activity during pregnancy. In the United States, only 15.8% of pregnant women vs. 26.1% of non-pregnant women reported engaging in the recommended physical activity guidelines [7]. This figure was even lower in a study from Brazil, where only 4.7% of pregnant women were physically active [8]. Only one-fifth of pregnant women in Ireland met the recommended guidelines and over 10% reported no physical activity [23]. Furthermore, a study examining lifestyle changes using the Pregnancy Risk Assessment Monitoring system (PRAMS), Ireland found that adherence to physical activity guidelines of moderate intensity activity was low (12.3%) but was particularly low for pregnant women with a body mass index > 25 kg/m^2^ (6.4%) [24]. A cross-sectional study carried out in Danish women who wore a pedometer for at least 5 days, found that mean footsteps were higher among normal-weight women compared to obese women [25]. Furthermore, a decline in physical activity in pregnancy was found in a study carried out in 305 overweight or obese women [26]. These low rates of physical activity during pregnancy, particularly for overweight and obese women, are concerning given the significant health benefits for both mother and baby [12].

Previous research on clinical effects of lifestyle interventions in overweight and obese pregnant women has shown conflicting results [27–31]. These results have been attributed to poor study design, lack of power, lack of consistency in terms of the target behaviour, and failing to identify the psychological determinants and behavioural mechanisms by which the intervention is expected to have an effect [32, 33]. These complex lifestyle interventions have consisted of interacting components including dietary and physical activity counselling, monitoring of weight and group exercise sessions or have been designed to prevent excessive gestational weight gain (GWG) and reduce the risk of GDM [34]. Other interventions include individual counselling sessions on weight control and motivational interviewing [35, 36]. Most of these studies have examined the combined effect of physical activity and dietary advice and guidance. Three randomized controlled trials (RCTs) [31, 37, 38] that assessed the isolated effects of exercise in pregnancy on GWG and clinical outcomes in overweight and obese women found no significant difference in GWG between exercise and control groups. However, a recent meta-analysis [39], found that structured physical exercise programs during pregnancy do decrease the risk of GDM. Future research needs to address these conflicting results, hence, there is a need to establish the potential effects of physical activity on clinical indicators, especially in overweight and obese pregnant women.

Using theory to identify the determinants of behaviour can increase the likelihood that an intervention will be effective [40, 41]. A systematic review [42] examining the determinants of physical activity during pregnancy found that intention to exercise, self-efficacy and barriers such as lack of time and tiredness were strong predictors of exercise. Moreover, a systematic review that evaluated the content of physical activity interventions in pregnancy found theoretically developed interventions were more likely to help reduce the decline of physical activity throughout pregnancy [43]. Therefore, more attention should be placed on using theory to identify perceived determinants of behaviour and barriers to physical activity behaviour in pregnancy in order to develop effective interventions.

**Fig. 1:**
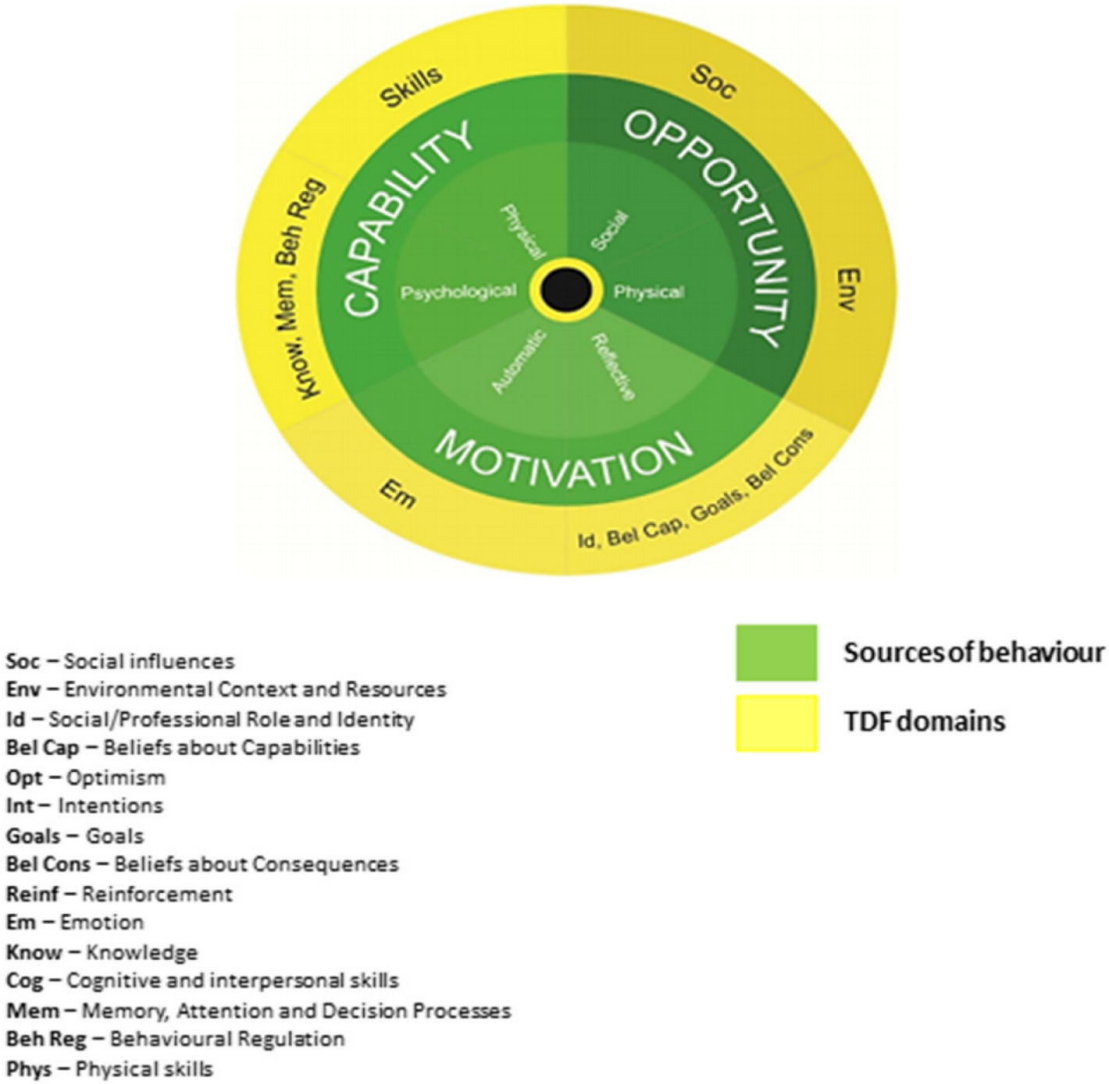
Theoretical domains framework an elaboration of the COM-B model. Reproduced with permission from Michie et al. [45, 46]

Health psychology offers theories of behaviour that can be used in maternity care interventions to help women make changes to lifestyle behaviours [34, 43, 44]. Michie and colleagues developed a framework derived from 33 commonly used behavioural theories and 128 psychological constructs called The Theoretical Domains Framework (TDF). The TDF has been identified as a useful tool for identifying determinants of behaviour and barriers to behaviour change. The TDF is an elaboration of the COM-B model which stands for “capability”, “opportunity”, “motivation” and “behaviour” [45, 46](Fig. 1). The COM-B model proposes that for any behaviour to occur a person must have the psychological and physical capability to perform the behaviour; the physical and social opportunity to engage in it and must be motivated to do so. Furthermore, when little is known about the population, qualitative research is useful to develop a theoretical understanding of the target behaviour [47–50]. To date, a number of empirical studies have used either the TDF or COM-B in order to develop behaviour change interventions in different contexts [51, 52] but to our knowledge this has not yet been done for physical activity in an overweight and obese pregnant population.

Therefore, the aim of this study was to use the TDF and corresponding COM-B model to identify enablers and barriers to physical activity in overweight and obese pregnant women, and to use this information to inform the development of an antenatal lifestyle intervention to improve physical activity levels during pregnancy.

## Method

### Study design

A qualitative approach was used. Semi-structured interviews were conducted with a sample of overweight and obese pregnant women at risk of GDM. Ethical approval was obtained from the University College Cork Clinical Research Ethics Committee of the Cork Teaching Hospitals (ref: ECM 4 (y) 06/01/15).

### Sampling and recruitment

Medical chart review identified a purposive sample of pregnant women with a body mass index (≥25 kg/m^2^) recruited during pregnancy from a public antenatal clinic at Cork University Maternity Hospital (CUMH). CUMH is a large academic maternity hospital in the South of Ireland where approximately 6657 new obstetrics patients entered in 2015 [53]. Eligible participants were approached individually and informed about the study by the attending midwife and researcher on site at their antenatal appointment. They were also provided with an information leaflet explaining the purpose of the study, how to participate and offered a small monetary compensation for participation. A €20 ‘One for All’ voucher for a local shopping centre was posted to each woman who participated once the interview had been completed. Simultaneously, a sub-study examining diet and physical activity behaviours in pregnant African women led by researcher (AEA) was on-going. These women were recruited from the same antenatal clinic, during the same period using the same sampling criteria and interview guide. Therefore, interview data on physical activity for these women were included in this analysis. Data on age, nationality, body mass index (BMI) and gestational age were recorded from medical charts where possible. GDM, employment status and miscarriages were recorded only for those women who reported them spontaneously during the interview.

### Interview process

Written informed consent was obtained from all participants at the start of the interviews. Face-to-face interviews were carried out in the antenatal clinic in CUMH on a day and time suitable for the participant by two researchers (CF) and (AEA) between June and September 2015. A semi-structured interview schedule was developed based on existing literature [34, 43, 54–56] and was used to facilitate the discussion (see Table 1). It consisted of open-ended questions and prompts about current lifestyle behaviours (physical activity and diet), challenges to engaging in healthy lifestyle and support mechanisms available. The interview schedule and process were piloted by interviewing two pregnant women at University College Cork. Following this pilot, additional probes and prompts were included to further explore women’s experiences in terms of weight management and lifestyle changes. Pilot interviews were not included in the final sample as the women were not eligible for inclusion in the study.

**Table 1:** Interview schedule used to facilitate the interviews

	Questions	Prompts/Probes
Intro	Tell me a little about your home life?	• First pregnancy? • Married, single? • Other Children – how many? • Employed – how many hours you work?
	Tell me a bit about your lifestyle at the moment?	• Diet – cravings, nausea • **PA** – active before pregnancy, frequency, duration • Have diet/**PA** patterns changed since pregnancy? • In what way and why?
Health	Has a HCP made you aware of the risks surrounding your pregnancy	• Excessive weight gain • GDM • Potential difficulties during delivery • How does that make you feel?
PA and Diet	What **PA** do you/would you like doing?	• Walking, running, exercises tailored for pregnancy, sports, gym?
	How important do you feel exercise and **PA** is during pregnancy?	• Fitness level • Mobility • Give you more energy • Help sleep
	Tell me what you think would be the best way to encourage women to be watchful of diet and **PA** during pregnancy?	• Through friends, other pregnant women, GP, nurses, information sessions, individual or group, exercise and diet programmes
Behaviour Change	Have you been given advice about dietary habits and **PA** since you became pregnant?	• HCP, family, friend, book, internet? • When was this? • How did you feel about the advice?
	What to do think are the main challenges to **PA** and diet changes during pregnancy?	• Lack of information/ support/ time/ resources
	Would you be interested in using technology to help you track and improve you **PA** and diet	• Mobile phone apps, text message/phone, web based information forums, pedometer? • Would these support mechanisms be useful? • If it provided you with information as well • If it provided you HCP with your information
	How would you feel about participating in a study where technology would be used as encouragement to increase **PA**?	• Mobile phone apps, text message/phone, web based information forums, pedometer • Access to internet, mobile phone
	Is there anything I haven’t asked you today you would like to mention?	

*PA* Physical activity, *HCP* Health care professional, *GDM* Gestational diabetes mellitus

### Data analysis

Interviews were recorded and transcribed verbatim. NVivo software was used to facilitate data analysis. Data analysis followed a framework approach [57]. An inductive thematic analysis was conducted to identify new emerging themes and to investigate a priori objectives using the TDF and COM-B model. Each transcript was read and re-read numerous times by the researcher (CF). Transcripts were coded line by line and analysed to identify similarities and differences. Following open-coding, broader categories were mapped onto the domains of the TDF and then, directly onto the six components of the COM-B model identifying emerging themes relating to enablers and barriers to physical activity. See Table 3 for description of the TDF domains and components of the COM-B model. All transcripts were coded by the researcher (CF) and a subset of interviews were independently coded and analysed by a second researcher (SMH). Minor differences arose in relation to the mapping of codes to the TDF domains, particularly when codes mapped to more than one domain. Differences were resolved by consensus involving a third researcher with expertise in using the TDF and COM-B model (MB) on one occasion, as some themes were coded into multiple TDF domains. Specifically, the domain of “behavioural regulation” and “goals” were merged due to the overlapping theme of action planning. Recruitment continued until new issues ceased to emerge and saturation occurred across the theoretical domains. Two further pregnant women were interviewed to check if any new themes emerged.

## Results

### Participants’ characteristics

In total twenty two overweight and obese pregnant women were interviewed. Data saturation occurred at interview twenty, as subsequent interviews did not contribute to the development of new themes. Eight interviews were included from the sub-study giving the overall sample of thirty overweight and obese pregnant women. Table 2 provides details of the participants’ characteristics including age, nationality, BMI and gestational age. GDM, employment status and miscarriages were only recorded if mentioned by the woman during the interview.

**Table 2:** Profile characteristics of participants (*N* = 30)

Nationality	
Chinese	2
French	1
Hungarian	1
Lithuanian	1
Irish	16
Nigerian	5
Sudanese	2
Congolese (Democratic Republic of Congo)	1
Ghanaian	1
Age	
20–29	6
30–39	14
40+	1
Unknown^a^	9
Gestation	
First Trimester (0 to 13 Weeks)	1
Second Trimester (14 to 26 Weeks)	8
Third Trimester (27 to 40 Weeks)	20
Not stated	1
BMI (kg/m2\)^b^	
Overweight 25–29	12
Obese ≥30	12
Unknown^c^	6
Pregnancy	
Singleton	29
Twins	1
Employment	
Working full time	10
Working part time	2
Out sick from work	2
Not working	6
Not stated	10
Gestational Diabetes Mellitus^d^	
GDM	5
Not stated	25
Miscarriages^e^	
Miscarriages	8
Not stated	22

^a^Not recorded from medical chart

^b^BMI taken from medical chart (calculated at booking visit by midwife)

^c^Midwife identified women as overweight and obese from chart but did not record BMI

^d^Only 5 women mentioned having gestational diabetes

^e^Only 8 women discussed having one or more miscarriages

### Physical activity clusters identified in pregnancy

**Fig. 2:**
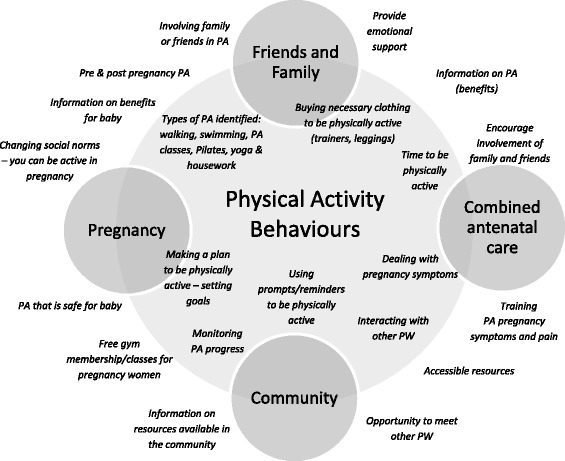
Physical activity clusters identified from the pregnant women interviews

From the open coding of the interview data, pregnant women identified a number of factors surrounding physical activity in pregnancy. Given the importance of physical activity during pregnancy and in order to highlight pregnant women’s perceptions, these different factors were categorised into four clusters that focus around friends and family, pregnancy, antenatal care and the community. These clusters are summarised in Fig. 2. Participants discussed different types of physical activity in pregnancy, the resources available and how family and friends could provide an important supportive role in physical activity participation. Participants also described the context in which these physical activity behaviours occur. Certain factors identified within these clusters are also present in the TDF and COM-B analysis, see results below. The main type of physical activity identified by the pregnant women includes walking, swimming, pilates, yoga and physical activity classes tailored for pregnancy.

### Summary of the TDF and COM-B model: Barriers and enablers to physical activity

Twenty one themes were identified that mapped directly onto ten of the TDF domains and the six COM-B components. The ten TDF domains included “skills”, “knowledge”, “behavioural regulation”, “goals”; “environmental context and resources”, “social influences”, “social/professional role and identity”, “beliefs about capability”, “intentions”, and “emotion”. The TDF domains not relevant to the context of physical activity in overweight and obese pregnant women were “optimism”, “reinforcement” “memory” and “belief about consequences”. These findings are described in greater detail below using the TDF and corresponding COM-B model (Table 3).

**Table 3:** Mapping of themes to the TDF domains and COM-B model

Themes	TDF	COM-B
- Fitness level prior to pregnancy - House work as a form of PA - Medical conditions and pregnancy symptoms (pain/energy/tiredness)	Knowledge (awareness of the existence of something: knowledge of condition)	Psychology capability Knowledge or psychological skills, strength or stamina to engage in the necessary mental process
- Limited knowledge surrounding PA benefits, types of PA in pregnancy and PA resources - Pregnant women discussed concerns around having that ‘conversation’	Knowledge (awareness of the existence of something: knowledge of condition)	Psychology capability Knowledge or psychological skills, strength or stamina to engage in the necessary mental process
- Self- monitoring, use of pedometer/step count/phone apps	Behavioural regulation (managing or changes action – self monitoring)	
- Women expressed interest in goal setting	Goals^a^ (mental representations of outcome or end states, that an individual wants to achieve)	
- Pregnant woman’s situation (family life/children/work/pets) - Financial situation - Weather/ built environment and resources within the community	Environmental context and resources (persons situation or environment)	Physical Opportunity Opportunity afforded by the environment involving time, resources, location, cues physical affordance
- Acknowledged support from family members, partner and friends - Interaction with other pregnant women [PA classes] was mentioned	Social influences (Process that can change thoughts feelings or behaviours – social pressure)	Social opportunity Opportunity afforded by interpersonal influences, social cues and cultural norms that influence the way we think
- ‘Every pregnant women is different’ - Differences in pregnancies	Social role and identity (set of behaviours and displayed personal qualities in a social or work setting)	Reflective Motivation Reflective process involving plans (self-conscious intentions) and evaluations (beliefs about what is good and bad)
- Using pregnancy as an ‘excuse’ - Concern for health of the baby - Feeling responsible - Difficulty breaking habits/mind-set	Beliefs about capability (acceptance of the truth, reality or validity about an ability, perceived behavioural control,, self-esteem, confidence)	
- Post-partum intentions (planning weight loss/healthy lifestyle)	Intentions (A conscious decision to perform a behaviour)	Reflective Motivation Reflective process involving plans (self-conscious intentions) and evaluations (beliefs about what is good and bad)
- Feelings of worry, concern and guilt during pregnancy - Fear based on previous pregnancy outcome/miscarriage	Emotion (complex reactions - fear, anxiety, affect, stress, depression, positive and negative effect, burn out)	Automatic Motivation Automatic processes involving emotional reactions, desires(wants and needs) impulses inhibitions drive states and reflex responses

^a^Behavioural regulation and goals were merged due to the overlapping construct of ‘action planning’

TDF domain not identified: optimism, reinforcement and belief about consequences

### Capability

#### Physical skills

In terms of the domain “physical skills”, pregnancy related symptoms were a common reason given by participants for undertaking little or no physical activity. These included muscle pain, pelvic or lower back pain, swelling and other conditions.

*‘The problems I had just stopped me [PA]. Like I got a polyp…which was heavy bleeding and the more I strained the body, even just a swim it was just like there was more pressure on it so I just said it was better to cut everything’ (Participant 15; 32 weeks pregnant)*

Furthermore, women who knew their pregnancy was high risk, decided themselves, that it was best not to engage in physical activity.

*‘I’m a high risk pregnancy so I couldn’t do any of the exercise then on this pregnancy. And then I have factor 5 blood so really clotting and all that, I have to take it easy’ (Participant 05; 28 weeks pregnant)*

Another barrier was that of feeling too tired to engage in physical activity; finding it hard to move, lack of energy and being physically drained.

*‘It’s harder to move faster now that I am pregnant. Like sometimes I have energy and some days I don’t… It’s difficult, like you feel like you want to do stuff but you can’t, your body is just tired and drained physically’ (Participant 20; 28 weeks pregnant)*

However, some women felt that physical activity during pregnancy did benefit them (e.g. helped them wake up, gave them energy and made them feel good). Likewise, being physically fit before pregnancy was identified as an enabler; if a woman was active before pregnancy she was more likely to keep it up.

*‘I don’t know I think it depends on everyone’s circumstances. Like a lot of women would be fit before they got pregnant and they would keep up their walking or running’ (Participant 01; gestation unknown)*

House work emerged as an enabler particularly for women who did not like exercise. These women considered household activities as part of their daily activity.

*‘No I wouldn’t get out and walk or anything like that…housework would be my activity during the day’ (Participant 04; 28 weeks pregnant)*

*‘Not really, there’s nothing really, I’m not a big fan of exercise. I will do the house work, the cleaning and the cooking’ (Participant 17; 36 weeks pregnant)*

### Knowledge

When considering the domain of “knowledge” there was concerns about safety and types of exercise appropriate in pregnancy.

*‘To be honest, I’m not good in what physical activities a pregnant woman should do because nobody really has told me about the kind of exercise you should be doing’ (Participant 28; 32 weeks pregnant).*

*‘I mean I don’t know can you do certain exercises so I would be worried that I could pull a muscle so I would be extra cautious I suppose at the gym cause I’m afraid and I wouldn’t really know’ (Participant 13; 32 weeks pregnant)*

These doubts were partly due to the limited information they reported receiving from their midwife or health care professional. This information was described as a *‘limited’*, *‘quick’*, *‘automatic’*, ‘*like a checklist’* and women felt the benefits of physical activity was rarely discussed.

*‘It’s very limited really, very limited. It’s a quick one minute conversation really in relation to it [PA/Diet]….I suppose nobody really sits you down to go through the implications of that or the benefits and stuff like that’ (Participant 21; 26 weeks pregnant)*

Furthermore when discussing ‘the conversation’ women felt more emphasis was placed on the clinical aspect of the visit rather than information and advice.

*‘They don’t tend to offer any advice good or bad in terms of weight management and activity and stuff like that. It’s more the blood pressure, checking the baby and stuff like that’ (Participant 21; 26 weeks pregnant)*

Some women felt that midwives assumed because they had other children they already had knowledge and information around being physically active in pregnancy.

*‘…what I found different was when they know that you have children already they kind of thinking that you know everything which is not true…you may forget, years apart, like between now and the last time I had a baby there is a three year gap so I can’t remember everything but they seem to assume because you have had other children you know already what to do’ (Participant 28; 32 weeks pregnant)*

Women actually felt less confident in terms of what they knew about physical activity and would have preferred more advice from their midwife.

*‘there’s no such thing as really showing you or describing it you know, or making sure that you are doing it [PA], I think that could be discussed or checked a little bit more’ (Participant 14: 30 weeks pregnant)*

Some women were active when they had “knowledge” of the health benefits (e.g. keeping muscles strong for labour). Furthermore, women expressed interest in attending pregnancy exercise classes; if they were provided with information on these classes in their area they would be more likely to attend.

*‘I think that would be a good idea [PA information & resources], like if you were given like numbers and sort of classes around that area at your clinic appointments for like types of yoga and stuff like that’ (Participant 04; 28 weeks pregnant)*

### Behavioural regulation and goals

In terms of “behavioural regulation” women’s comments on technology suggested that action planning and self-monitoring would be an enabler to physical activity. When discussing technology, women explained that a ‘pedometer’ or ‘step count’ might help in terms of motivation and to monitor current levels of physical activity.

*‘If there was definitely some sort of measurement like a pedometer or something like that, just something that would flag where you are at and what your targets should be’ (Participant 21; 26 weeks pregnant)*

Some women suggested setting “goals” as an enabler to physical activity, providing them with targets to accomplish.

*‘I am very goal driven, I would love that, if someone said ' you need to walk three miles this week and you need to do four laps of the pool and something else, you know you would hit your targets and you know then that even if they say that was helping you, that you are going a good job. You’re doing something good anyway’ (Participant 18; 14 weeks pregnant)*

Although women felt a pedometer or step count would help with motivation, other forms of technology did not have the same perceived benefit. Women disliked the idea of tracking physical activity (number of days, length of activity time) in a phone app if it was linked with the antenatal clinic. They felt like ‘*big brother*’ would be watching or that it was a chance for their health care professionals to *‘check up on me’* calling it an *‘invasion of privacy’*. Furthermore some women felt that tracking physical activity would be a *‘burden’* or like *‘homework’* and that with their busy lifestyles they would just forget.

*‘I’m not actually that good of keeping track of anything really like that [PA] (laughs) I would try to write things down but I would just be so busy or I would forget and I wouldn’t do it, so I wouldn’t be a good user of those [pregnancy apps]’ (Participant 13; 32 weeks pregnant)*

### Opportunity

#### Environmental context and resources

Women’s opportunity to engage in physical activity in pregnancy was often hindered by work and family commitments. Even though they were motivated to be physically active, often constraints in the way of time and bad weather conditions justified not participating in physical activity.

*‘I suppose prior to the first pregnancy I could go from work to exercise and then come home. Whereas, now if I do that I don’t see my son before he goes to bed. So I just can’t fit it into my day to be honest, it’s more challenging’ (Participant 21; 26 weeks pregnant)*

Some women identified a lack of financial means as well as a lack of targeted services specifically tailored for pregnancy as barriers to physical activity. Women suggested subsidised services as a solution to financial difficulties. Making services *‘financially viable’* might encourage the use of a gym or exercise class’s thus enabling physical activity.

*‘I mean I’m not going just because I have two kids I have a massive big mortgage and I actually can’t afford the full membership to go swimming…….Free gym membership for pregnant woman for 9 months (laughs) that would be great, even I would go then (laughs)’ (Participant 16; 38 weeks pregnant)*

### Social influences

A commonly reported enabler was that of “social influences” which included family and friends encouragement of physical activity. Women’s partner or husbands were the most influencing factor (e.g. *‘always pushing me to go for a walk’*, *‘he would drag me out for a walk’*).

The women’s husbands were not seen as a barrier to PA while other family members were.

*‘Put your feet up' that’s what I get especially over the last four weeks, from my mother in law’ (Participant 16; 38 weeks pregnant)*

Women also expressed an interest in pregnancy physical activity classes giving mothers a chance to *‘talk’* comparing it to a *‘support group’*.

*‘…it would be that extra motivation [PA classes]. Get out and make friends and talk more, and enjoy the activity more’ (Participant 04; 28 weeks pregnant)*

### Motivation

### Social role and identity

A clear justification for not engaging in physical activity was the ‘individual’. It was commonly reported that *‘every woman is different’* and *‘every pregnancy is different’* and it was up to that ‘individual’ whether or not they would make healthy choices or be physically active.

*‘I think it definitely depends on the individual, I think it depends on the pregnant mother whether they want to be healthy or not…’ (Participant 01; gestation unknown)*

### Belief about capability

When considering “belief about capability” pregnancy was viewed as a time for change particularly for the benefit of the baby ‘*I just have to… be as healthy as I can be now, I mean it’s all for the baby’ (Participant 13; 32 weeks pregnant)*. The foremost feelings that prevailed throughout the interviews were the sense of *‘responsibility’* in providing the best for the baby in terms of healthy lifestyle behaviours.

*‘…every woman is different and every woman will take on board information differently* [diet & PA]*. I think it is very important when you’re pregnant, you need to just take responsibility like, and you do. (Participant 19; 27 weeks pregnant)*

Some women also described how they were changing behaviour to be healthy not only for the baby but for themselves.

*‘..when I came out of my doctor I knew I was going to do something that was going to help me and the baby and that my actions would make us healthier together ya know. (Participant 18; 14 weeks pregnant)*

At the same time, pregnancy provided a reason to not make healthy changes (e.g. *‘…like sure I’m pregnant. I’m going to be big anyway’ (Participant 09; 39 weeks pregnant)).* Woman felt that pregnancy could be used as an *‘excuse’* and that *‘mind-set’* played a big part in whether or not you would make any changes. Some women stated they would have to have been physically active at the start of pregnancy in order to keep it up and that breaking bad habits in pregnancy is difficult.

*‘No I would have to have been doing it from the start [PA]. I wouldn't have picked it up half way through. I definitely would have had to have started at the beginning. I mean I told myself at the start, I actually wouldn't mind doing that [PA] and keeping it up but I just didn't and then I just stopped and sat and eat….it’s hard to break that habit especially when you are pregnant as you do use it as an excuse’ (Participant 02; gestation unknown)*

### Intentions

Others reported being motivated when talking about after pregnancy and their implicit intentions to change (e.g *‘I have it planned out in my head’*).

*‘I know I am not having any more and I tell myself afterwards I’ll get back into it’ (Participant 02; gestation unknown)*

*‘So I said right when this baby now is done…after I have recovered I’m going back to my [PA] classes’ (Participant 05; 28 weeks pregnant)*

### Emotion

In terms of “emotion”, enablers to physical activity included feelings of *‘guilt’* and *‘concern’*.

*‘if I could get away with it [no PA], if I could I would definitely but I know I would feel pure guilty. I know I would have them [health care professionals] looking at me and I would feel fierce guilty’ (Participant 18; 14 weeks pregnant)*

‘…*the first time round I could go for walks, I was taking care of my health and ya know, you kind of that bit worried the first time round, you make sure you are doing the best for the baby and yourself’ (Participant 01; gestation unknown)*

A fear based on previous pregnancy outcomes was highlighted with women afraid to do anything in pregnancy due to previous miscarriage experiences.

*‘…from the moment I knew I was pregnant it has been terrifying for me. Because like I’m after having 3 miscarriages in 2 years it’s not a nice thing to experience, I mean you’re constantly waiting to see that heartbeat..’ (Participant 05; 28 weeks pregnant)*

## Discussion

The aim of this study was to systematically identify the barriers and enablers to physical activity for women who are overweight and obese in pregnancy using the TDF and COM-B model. A wide range of barriers and enablers were identified which influenced women’s capability, motivation and opportunity to engage in physical activity with women providing more information about barriers than enablers.

In the current study, the most commonly reported barrier to physical activity during pregnancy was “knowledge”. It was clear from the findings that women were unclear on what types of physical activity they could engage in while pregnant and whether physical activity was safe. This finding is similar to that of a qualitative study conducted in the US, in which pregnant women mentioned a lack of advice regarding physical activity [58]; the most information they received from their midwives was to ‘carry on as usual’ [59]. Perhaps this lack of information can explain why adherence to physical activity guidelines is so low particularly for pregnant women with a BMI > 25 kg/m^2^ (6.4%) [24]. Health care professionals are key to enhancing pregnant women’s knowledge of being physical active and the benefits of being active in pregnancy [60]. Furthermore, many women received little or no advice on appropriate weight management in pregnancy. Service providers [61], similar to the women here, considered verbal advice offered to women on topics such as lifestyle and weight management to be inconsistent and unsupported by written information [62]. This is perhaps not surprising given the lack of Irish guidance regarding weight management in pregnancy [22]. However, despite this the women actually expressed little concern about weight gain.

“Physical skills” such as pregnancy-related symptoms (e.g. morning sickness/nausea/pelvic pain) were common barriers to physical activity. However, research has shown that being physical active in early pregnancy can reduce these symptoms [11, 12]. Thus this information may be a useful motivational strategy to encourage overweight and obese women to be active early on. Furthermore, high risk pregnancies were identified as a barrier, yet, research has indicated that in the case of risk factors for preeclampsia, exercise has been seen to promote maternal circulation, improve maternal fetal vascularity and boost the immune system of women [63]. For women with high risk pregnancies, physical activity is recommended with some restrictions; but there are currently no clear recommendations available [64], therefore, evidence based guidelines are required for health care professionals in order for them to guide women about safe activity in pregnancy given their health status. Another barrier reported by the women was tiredness and a lack of energy due to being pregnant, work and family commitments. This is consistent with previous literature, feeling tired or having no energy are the most commonly reported reasons for not being active [58, 65–67].

The women identified “social influences” indicating the relative importance of advice received from family and friends in initiating physical activity behaviour. Also, the women enjoyed meeting other pregnant women and expressed interest in physical activity classes tailored for pregnancy. Healthcare professionals need to take a holistic approach to care, taking into consideration the women’s social support network and influences to include their partners in group pregnancy sessions. Action planning and goal setting were identified by the women as a means of motivation and that pedometers and step counts could help with self-monitoring. A review, examining the use of pedometers to increase physical activity and improve health, concluded that pedometers were associated with significant increases in physical activity in an adult population [68]. Furthermore, in a study with pregnant women the pedometer was acceptable to the women [25]. Thus, future interventions should include some component of self-monitoring in order to improve physical activity levels in overweight and obese pregnant women.

Analysis using the TDF provided a detailed understanding of the barriers and enablers to physical activity for pregnant women and the refinement of the findings into the COM-B model has set the stage for developing a theory and evidence based intervention to increase physical activity levels in overweight and obese pregnant women. Using these frameworks added substantial strength to this study because it is composed of theoretically derived domains based on a comprehensive list of behavioural theories. This will help to identify potentially relevant domains and to select a set of relevant theories to investigate the target behaviour in depth at a later stage. While the study has some clear strengths, there were some potential limitations. While the TDF provided a comprehensive framework for understanding types of enablers and barriers to physical activity among this population, at times it was difficult to categorise themes due to lack of clarity in the definitions of the theoretical domains. Where this happened, the best solution was determined through discussion with members of the research team (CF) and (SMH). An additional limitation was the sampling frame for the study; all women were recruited through a public clinic in one maternity hospital setting potentially limiting diversity in study findings. Furthermore, even though this ethnically diverse sample of pregnant women shared similar views regarding physical activity, research is warranted to assess racial or cultural differences in overweight and obese pregnant women.

## Conclusion

This research provides an important overview of the behavioural factors enabling or inhibiting physical activity and has also identified a system of behaviours that may be relevant in order to increase physical activity levels amongst overweight and obese pregnant women. Using the TDF and COM-B model is a theoretical starting point for understanding behaviour within specific contexts and to make a ‘behavioural diagnosis’ of what needs to change to alter behaviour. The COM-B model forms the hub of the Behaviour Change Wheel (BCW) which provides a systematic and transparent way to conduct a behavioural assessment, identify the target behaviour, select intervention functions and to develop theory based intervention strategies [45]. The findings suggest a lack of knowledge around safe types of physical activity in pregnancy and awareness of the potential benefits for mother and baby. Interventions which provide continuing support from health care professionals and involve partners and family members are potential approaches to consider for interventions in pregnancy. In future research, we will use the behaviour change wheel to identify intervention functions to systematically develop a lifestyle intervention to increase physical activity levels for overweight and obese pregnant women. Developing an antenatal intervention that targets these salient barriers to physical activity will have greater potential to change behaviour.

## Additional file

Additional file 1 **Table S1.** Coding frame: barriers and enablers. (DOCX 38 kb)

## Abbreviations

BCW: Behaviour Change Wheel
COM-B: Capability, opportunity and motivation-behaviour
PW: Pregnant woman
TDF: Theoretical Domains Framework
