# Supplementary material for: Enablers and barriers to physical activity in overweight and obese pregnant women: an analysis informed by the theoretical domains framework and COM-B model
Source: BMC Pregnancy Childbirth. 2018 May 21;18:178. doi: 10.1186/s12884-018-1816-z (PMC5963099; doi:10.1186/s12884-018-1816-z)
Supplement: Supplementary file 1 — Table S1. Coding frame: barriers and enablers. (DOCX 38 kb) [file 12884_2018_1816_MOESM1_ESM.docx]

**Table S1: Coding frame – Barriers and enablers to physical activity for overweight and obese pregnant women**

| **COM-B component** | **Relevant TDF domain that map onto the COM_B** | **Codes mapped to TDF framework** | **Example of quotation (s) Enablers** | **Example of quotation (s) Barriers** |
| --- | --- | --- | --- | --- |
| **Physical capability –** physical skill, strength, stamina | **Physical skills** | - Fitness level prior to pregnancy - House work as a form of PA - Medical conditions and pregnancy symptoms (pain, energy, tiredness) | Benefit: *‘I would go back to swimming. It makes you feel so much lighter. It’s great to feel that way’ (PW16)*  Benefit: ‘*It’s just enjoyable and helpful [aqua aerobics], that’s probably why I didn’t feel so pregnant. I didn’t have any back issues, all my muscles were working and you concentrate on your breathing as well’ (PW16)*  Benefit: *‘Like it actually wakes me up….I do get energy after it, like if I go from my walk, I am well able to clean the house whereas it I don’t go for my walk I am just thrown there’ (PW15)*  Benefit: *‘A couple of days ago I was exhausted and I was just like no, I should just get out and get some air and it was actually so much better to get out, I felt so much better than sitting around the house, it’s good for your mental health as well. Definitely’ (PW13)*  Benefit: *‘Swimming, I would love to do more swimming because it takes the pressure off the bump. I had fierce pressure. Even with the pelvic girdle pain if I stayed in the pool it took the pressure off’ (PW18)*  Benefit: *‘I always feel good after a walk, like I wouldn’t go for a long’ (PW20)*  Housework: *‘No I wouldn’t get out and walk or anything like that….housework would be my activity during the day’(PW04)*  Housework: *‘Not really, there’s nothing really, I’m not a big fan of exercise. I will do the house work, the cleaning and the cooking’ (PW17)*  Fitness: *‘I don’t know I think it depends on everyone’s circumstances. Like a lot of women would be fit before they got pregnant and they would keep up their walking or running’ (PW01)* | Medical: *‘I do go for a walk once in a while, because of the pelvic pain it is very difficult for me I feel pain every time I go for walk so I don’t go all the time’ (PW25)*  Medical: *‘I suppose I could walk a bit more. You see I’m getting physio at the moment…I have SPD. I have Pain in my pubic area. So I supposed to do fewer activities’ (PW04)*  Medical: *‘I did have a little bit of swelling in the feet after work and you are coming and resting , its adjusting’ (PW14)*  Medical: *‘The problems I had just stopped me [PA]. Like I got a polyp…which was heavy bleeding and the more I strained the body, even just a swim it was just like there was more pressure on it so I just said it was better to cut everything’ (PW15)*  Medical: *‘I’m a high risk pregnancy so I couldn’t do any of the exercise then on this pregnancy. And then I have factor 5 blood so I really clotting and all that, I have to take it easy’ (PW05)*  Energy: *‘It harder now to move faster now that I am pregnant. Like sometimes I have energy and someday I don’t… It’s difficult, like you feel like you want to do stuff but you can’t, your body is just tired and drained physically’ (PW20)*  Energy: *‘Getting on the cross trainer but after a half hour I was just like, I was pretty exhausted and the next day was pretty bad, my energy levels just aren’t what they used to be’ (PW13)* |
| **Psychology capability –** knowledge or psychological skills, strength or stamina to engage in the necessary mental process | **Knowledge** (Awareness of the existence of something – including knowledge of condition/ scientific rationale, procedural knowledge. Knowledge of task environment) | - Limited knowledge of PA benefits, types of PA in pregnancy and PA resources - Pregnant women discussed concerns around having that ‘conversation’ | PA knowledge: *‘I suppose being pregnant doesn’t stop you from doing exercises if you are used to doing exercise, you have to continue doing it but you have to know which one is good in your condition. This is what will be the challenge’ (PW28)*  PA knowledge*: ‘I think there is enough information* [PA], *people do give u information it’s just to, it’s just to go out and ehhhh (laughing) do it! I think once you start its ok but the thought of it is actually worse’ (PW08)*  PA knowledge*: ‘I suppose like yeah pregnancy is difficult and you would be tired, but it’s not a disease like (laughter), like I’d still continue to walk’ (PW09)*  PA benefits: *‘I think it’s important to be active of course to keep your muscles strong for the labour and everything’ (PW04)*  PA benefits: *‘If there is something light or some kind of pregnancy classes. Tailored for pregnancy, I think it would be great for people. It gets, it gets, it helps with your preparation for the birth and all that stuff’ (PW 05)*  PA resources: *‘I think that would be a good idea* [PA information & resources], *like if you were given like numbers and sort of classes around that area at your clinic appointments for; like types of yoga and stuff like that’ (PW04)*  The conversation: *‘I suppose it all about having the conversation, it about being given the right information especially around the first scan’ (PW19).* | PA knowledge: *‘And I even with the risk it’s just the nervousness that you don’t want to be doing physical activity or something like that. And then you’re scared of your life that you’re after doing something’ (PW05).*  PA knowledge: *‘I had just signed up for them* [classes] *and then I was, got pregnant. Then I was thinking can I do them. And I was thinking can I do the zumba but I was think there is too much jumping and dancing in that’ (PW05).*  PA knowledge: ‘*Like in the gym with other people sometimes it could be a bit dangerous cause people can be working out around you and might hit you so somewhere specially for pregnant women’ (PW03)*  PA knowledge: *‘I mean I don’t know can you do certain exercises so I would be worried that I could pull a muscle so I would be extra cautious I suppose at the gym cause I afraid and I wouldn’t really know what, like I think did ask at one stage like about what I should and shouldn’t be doing but she said do what you normally do but just take it easy (laughs) that’s the advice I got so like it’s not really helpful at all’ (PW13)*  PA knowledge: *‘Cause often when you in the gym on your own and you’re not really sure, like I know you’re not supposed to lift heavy weight unless you have a help especially if you’re not used to it but [am] the rowing machine, can you use than when you are pregnant?’ (PW13)*  PA knowledge: *‘To be honest, I’m not good in what physical activities a pregnant woman should do because nobody really has told me about the kind of exercise you should be doing’ (PW28).*  PA knowledge: *‘Like I know the services are fantastic and they’re brilliant and if there are any problems they are in there like a bullet out of a gun but I think it’s the smaller things that they oversee* [lifestyle information]*’ (PW18)*  PA knowledge: *‘what I found different was when they know that you have children already they kind of thinking that you know everything which is not true…you may forget, years apart, like between now and the last time I had a baby there is a three years gap so I can’t remember everything but they seem to assume because you have had other children you know already what to do’ (PW28)*  PA resources: *‘I have, I never really got information about classes around. I seem to have to seek it out myself. And I ended up never going to do that, you know what I mean’ (PW04)*  The conversation: ‘*All the little things, it was more a checklist than an actual conversation. Obviously it was like I need to discuss this, this, this based on whatever the chart or folder says but it was more of a checklist thing rather than a conversation’ (PW16)*  The conversation: *‘It’s very limited really, very limited. It’s a quick one minute conversation really in relation to it [PA/Diet]. I would have done a good bit more research myself, the first time round in terms of what to avoid and what’s important in terms of baby as well….I suppose nobody really sits you down to go through the implications of that or the benefits and stuff like that.(PW21)*  The conversation: *‘there’s no such thing a really showing you or describing it ya know, or making sure that you are doing, I think that could be discussed or checked a little bit more’ (PW14)*  Tech: *‘I feel the internet [for information] can be great but it can also be the worst thing in the world because your almost self-diagnosing yourself with every single issue that you might not even have so I do think it should be a little bit more relevant to the person’ (PW14)* |
|  | **Behaviour Regulation & Goals*** (managing or changes action – self-monitoring, breaking habits or action planning) | - Self- monitoring, use of pedometer/ step count - Women expressed interest in goal setting | Use of technology: *‘Like I hear of step counter for number of steps and stuff like that and I think that would be great, I think it would motivate you, you know’ (PW10)*  Use of technology: *‘I think pedometers are great, of course I don’t have one myself (laughs) but (amm) I think they are brilliant cause (amm) you know how far you have walked in a day and how many calories which is great’ (PW13)*  Use of technology: *‘If there was definitely some sort of measurement like a pedometer or something like that, just something that would flag where you are at and what your targets should be’ (PW21)*  Use of technology: *‘People who have an apple watch, they will get a buzz and it will tell them to stand up for a bit and I was like seriously but that’s the way it works. Reminding you to exercise and stuff like that….I mean technology, it has such a big place in our lives and it’s not the end, if we could do something it would be good but (emm) I don’t know it’s all about how you promote it and how visual and how people take to it’ (PW16)*  Use of technology: *‘If you can link it together, how much exercise you should do, what food, how many calories every day and you can track or like today I did less exercise so tomorrow I have to do the amount I should be doing’ (PW03)*  Goals: *‘I guess if you had a goal and you had it in your mind that would help you be healthier’ (PW14)*  Goals: *‘I am very goal driven, I would love that, if someone said ' you need to walk three miles this week and you need to do four laps of the pool and something else' you know you would hit your targets and you know then that even if they say that was helping you, that you are going a good job. You’re doing something good anyway’ (PW18)* | Use of technology: ‘*Maybe for some people it might, because it is kind of a big brother as well [tracking]. It wouldn’t be for me, but for some people it might be an issue. If you had weight problems, you know someone saying “keep a track of it”’ (PW27)*  Use of technology: *‘I could see how some people would think that like big brother is watching you and they might not like it (laughing)’ (PW08)*  Use of technology: *‘Check up on me [phone app tracking].....I wouldn’t like that, that invasion of privacy. If the person feels like they can’t be responsible for their pregnancy of course but you'd have to be completely stupid to not be able to do it yourself or take responsibility. You should be able to look after your own pregnancy’ (PW19)*  Use of technology: *‘I’m not actually that good of keeping track of anything really like that [laughs] I would try to write things down but I would just be so busy or I would forget and I wouldn’t do it, so I wouldn’t be a good user of those [pregnancy] apps’ (PW13)*  Use of technology: *Yeah if I was supposed to be doing it probably would be more of a burden, I’d be going “oh for god sake I’ve to sit down now and do that” rather than having it for your own personal, tracking I suppose…… if it was seen as more of a homework thing [tracking PA in app] I’d be less likely to use, do it’ (PW07)*  Goals: …*well I would say goals instead of targets anyways. I think targets might push it over the edge, I think we should use the words goals instead, so goals you have to try reach. I guess if you had a goal and you had it in your mind that I would help you be healthier than ya’* |
| **Physical Opportunity** – Opportunity afforded by the environment involving time, resources, location, cues physical affordance | **Environmental context and resources** (persons situation or environment – environmental stressors, resources, material resources, organisational culture, climate, salient events critical incidents, interactions, barriers and facilitators) | - Pregnant woman’s situation (family life/children/work/pets) - Financial situation - Weather/ built environment and resources within the community | Financial & resource: *‘I mean jokes aside anything financially viable. If you were to, I don’t know maybe because you are pregnant you pay half price or something like that so that people would go [gym]’ (PW16)*  Financial & resource: *‘Free gym membership for pregnant woman for 9 months (laughs) that would be great, even I would go then (laughs)’ (PW16)*  Pets: *‘…we have pets at home and we would take them for a walk, felt like we had too’ (PW01)*  Pets: *‘I have dog so I do take her out for a walk. Have to bring the little dog out [laughter]. Ya know a little westie’ (PW05)* | No time: *‘It’s very hard to just get time to go [swimming]. I find that anyway….when you are busy and you have commitments and stuff. I find it very tough to get out’ (PW18)*  No time: *‘I manage the home like and between everything I just don’t get around to it, it would stop me from being active. I never really have time to myself, like your trying to get things done for the baby, then you’re trying to involve the kids so that there not feeling left out, so it is very hard’ (Pw10)*  No time: *‘I suppose prior to the first pregnancy I could go from work to exercise and then come home. Whereas, now if I do that I don’t see my son before he goes to bed. So I just can’t fit it into my day to be honest, it’s more challenging’ (PW21)*  No time: *‘I don’t have any time as I work full, I leave my house at half seven in the morning and come back at five in the evening so it’s time to look after the children. There’s no really time for physical exercise’ (PW28)*  Financial: *‘I mean I’m not going just because I have two kids I have a massive big mortgage and I actually can’t afford the full membership to go swimming and that’s the only reason why’ (PW16)*  Resources: *‘I mean I’m from Killarney and there’s just not many for pregnancy [classes], like I like pregnancy yoga and stuff like that, I would like to try those things but there’s nothing really in the town’ (PW08)*  Resources: *‘I have always been looking out for them but with working full time it’s hard, these groups are like usually mid-morning like 11 on a Tuesday morning and they are never on a 7 on a Thursday evening so really I wouldn’t be able to go to them’ (PW13)*  Built environment: *‘I live in quite a hilly area so. It’s hard, obviously with the buggy and everything, It would be harder. If I want to go on the flat, you know have to get in the car then’ (PW07)*  Built environment: *‘I live in the country side on a busy road so it hard to go walking there and I don’t drive, its only my husband that drives and when he’s at work I can’t really go anywhere’ (PW08)*  Weather: ‘*If you have things ready for the weekend and doing PA and stuff it depends and weather and stuff like’ (PW01)*  Weather: *‘Summer time the weather is OK and we could probably go to the park every day but if it start raining you are stuck in the house for a couple of weeks so it’s not good’ (PW03)* |
| **Social opportunity –** Opportunity afforded by interpersonal influences, social cues and cultural norms that influence the way we think | **Social influences** (Process that can change thoughts feelings or behaviours – social pressure, social norms, group conformity, social comparisons, social support, group identity, modelling | - Acknowledged support from family members, partner and friends - Interaction with other pregnant women [PA classes] was mentioned | Husband: *‘Actually, that’s my problem because I hate it [PA], my husband is always pushing me to go for a walk’ (PW22)*  Husband: *‘My husband and I would be quiet active; we have a dog so me and my husband would go the beach at the weekend’ (PW13)*  Husband: *‘Probably my husband would be the one obviously, that I speak to more often about it. He would say, “Let’s try and get out for a walk tomorrow.” that kind of encouragement’ (PW21)*  Husband: *‘Especially my husband, he would drag me out for a walk, you need to move cause really when you get big and walk is really important’ (PW03)*  Family: *‘We [family] do try to do something physical like walking, walking is the main thing, trying to go to the park, trying to run around rather than doing something indoors’ (PW01)*  Family: *‘I do try to get my son involved in going swimming’ (PW01)*  Classes: *‘I think a lot of people would take that up [pregnancy PA classes]. Even if it was just something simple like in a community centre like, simple exercises just to help, I would definitely go’ (PW18)*  Classes: *‘I think a good idea would be, like I don’t live in cork city, I live in Clonakilty, I think if there was a pregnancy group that met once a week, where we could talk about diet and exercise and how we are doing and feeling, like a support group’ (PW13)*  Classes: *‘I’d say it would be that extra motivation [PA classes]. Get out and make friends and talk more, and enjoy the activity more’ (PW04)*  Classes: *‘I would love that, ya cause at least you know you doing it right and you won’t strain yourself and in case you get an injury [PA classes]’ (PW15)*  Classes: *‘I even find those parent and toddler groups, they are great. A way of kids mixing with other children, mothers to talk and for women living outside the city and I think that would be a great stepping stone, like let’s have a walking day’ (PW01)*  Friend: *‘So when I was going to the gym, my friend was going so we pushed each other so I think if I going with someone who sticks to it then I would’ (PW08)* | Family: *‘Put your feet up' that’s what I get especially over the last four weeks, from my mother in law’ (PW16)*  Family: *‘Walking with the kids is totally different on my own [laughter] it’s less stressful, not with the 11 year old now because she kind of does her own thing. The 3 year old, he doesn’t walk; he runs. So you’re constantly calling him back. And you’re watching then if you’re out on the road, like ya know if you’re on the footpath you’re constantly watching in case he goes on the road. So I try and go on my own if I can. But if not then I bring him with me like’ (PW10)* |
| **Reflective Motivation –** Reflective process involving plans (self-conscious intentions) and evaluations (beliefs about what is good and bad) | **Professional /social role and identity (set of behaviours and displayed personal qualities in a social or work setting )** | - The ‘individual’ | Every pregnancy is different / depends on the person (social role identify) |  |
|  | **Belief about capability** (acceptance of the truth, reality or validity about an ability, talent) | - Using pregnancy as an ‘excuse’ - Concern for health of the baby - Feeling responsible - Difficulty breaking habits/mind-set | Baby: ‘*I just have to…keep going and just be as healthy as I can be now, I mean it’s all for the baby’ (PW13)*  Baby: *‘I think it’s every women will make the choice herself [healthy lifestyle] and what she’s feels herself, what she needs for her body and the baby’ (PW12)*  Baby: *‘when I came out of my doctor I knew I was going to do something that was going to help me and the baby and that my actions would make us healthier together ya know. (PW18)*  Responsibility: *‘I think when a woman, no matter when pregnant or expecting pregnancy when you carry someone inside you, you should have the responsibility to provide as best as you can for the baby’ (PW03)*  Responsibility: *‘…very woman is different and every woman will take on board information differently. I think it is very important when you’re pregnant, you need to just take responsibility like, and you do. (PW19)* | Mind-set: *‘I don't know if it would have mattered, I can’t tell, because I just get my days where I don't care and I should care I know. I'm just not in the humour. (PW02)*  Mind-set: *‘If I was motivating myself more I suppose [PA] yeah that would encourage me, it’s just hard to get into that mind-set’ (PW09)*  Mind-set: *‘The first week you are eager to go [Gym], oh I’m going to gym but the following weeks maybe once in a week, in a month maybe once in a month before you know it you forgot all about it’ (PW30)*  Habit: *‘No I would have to have been doing it from the start [PA]. I wouldn't have picked it up half way through. I definitely would have had to have started at the beginning. I mean I told myself at the start, I actually wouldn't mind doing that [PA] and keeping it up but I just didn't and then I just stopped and sat and eat….it’s hard to break that habit especially when you are pregnant as you do use it as an excuse’ (PW02)* |
|  | **Intentions** (A conscious decision to perform a behaviour – stability of intentions, stages of change) | - Post-partum intentions (planning weight loss/healthy lifestyle) | Intentions*: ‘I have it planned out in my head that, I do evenings and I finish at half 1 and if I went to the gym for a half hrs. . I know it’s night time but I will try and I would be happy with that’ (PW02)*  Intentions*: ‘I know I am not having any more and I tell myself afterwards I’ll get back into it and I will stop all the rubbish eating and I know I will do it’ (PW02)*  Intentions*: ‘So I said right when this baby now is done…after I have recovered I’m going back to my classes’ PW05* |  |
| **Automatic Motivation** – automatic processes involving emotional reactions, desires(wants and needs) impulses inhibitions drive states and reflex responses | **Emotion** (a complex reactions (experiential, behavioural, physiological- fear, anxiety, affect, stress, depression, positive and negative effect, burn out) | - Feelings of worry, concern and guilt during pregnancy - Fear for baby based on previous pregnancy outcome/ miscarriage | Guilt: ‘…*if I could get away with it [no PA], if I could I would definitely but I know I would feel pure guilty. I know I would have then looking at me and I would feel fierce guilty’ (PW18)*  Worry: *‘I think no matter how babies or pregnancies the woman has for every single one they still worry, worried,, they want to make sure the baby is health’*  *(PW03)*  Worry: ‘…*the first time round I could go for walks, I was taking care of my health and ya know, you kind of that bit worried the first time round, you make sure you are doing the best for the baby and yourself’ (PW01)* | Fear: ‘...*from the moment I knew I was pregnant it has been terrifying for me. Because like I’ve, after having 3 miscarriages in 2 years it’s not a nice thing to experience, I mean you’re constantly waiting to see that heartbeat..’ (PW05)* |
| ****behavioural regulation and goals were merged due to the overlapping construct of ‘action planning’***  PA, Physical activity; HCP, Health care professional | | | | |
